# Cell proliferation in human epiretinal membranes: characterization of cell types and correlation with disease condition and duration

**Published:** 2011-07-02

**Authors:** Sarit Y. Lesnik Oberstein, Jiyun Byun, Diego Herrera, Ethan A. Chapin, Steven K. Fisher, Geoffrey P. Lewis

**Affiliations:** 1 The Academic Medical Center Amsterdam, University of Amsterdam, The Netherlands; 2 Neuroscience Research Institute and Department of Molecular Cellular and Developmental Biology, University of California Santa Barbara, CA; 3 Mayachitra Inc., Santa Barbara, CA

## Abstract

**Purpose:**

To quantify the extent of cellular proliferation and immunohistochemically characterize the proliferating cell types in epiretinal membranes (ERMs) from four different conditions: proliferative vitreoretinopathy (PVR), proliferative diabetic retinopathy, post–retinal detachment, and idiopathic ERM.

**Methods:**

Forty-six ERMs were removed from patients undergoing vitrectomy and immediately fixed in paraformaldehyde. The membranes were processed whole and immunolabeled with either anti-MIB-1 or anti-SP6 to detect the K_i_-67 protein in proliferating cells, in combination with anti-glial fibrillary acidic protein or anti-vimentin to identify glia, anti-ezrin to identify retinal pigment epithelial cells, *Ricinus communis* to identify immune cells, and Hoechst to label nuclei. Digital images were collected using a laser scanning confocal microscope. The cell types were identified, their combined proliferative indices were tabulated as the average number of anti-K_i_-67-positive cells/mm^2^ of tissue, and the number of dividing cells was related to the specific ocular condition and estimated disease duration.

**Results:**

ERMs of all four types were shown to be highly cellular and contained proliferating cells identified as glia, retinal pigment epithelium, and of immune origin. In general, membranes identified as PVR had many more K_i_-67-positive cells in comparison to those in the other three categories, with the average number of K_i_-67-positive cells identified per mm^2^ of tissue being 20.9 for proliferative diabetic retinopathy, 138.3 for PVR, 12.2 for post–retinal detachment, and 19.3 for idiopathic ERM. While all membrane types had dividing cells, their number was a relatively small fraction of the total number of cells present.

**Conclusions:**

The four ERM types studied demonstrated different cell types actively dividing at the time of removal, confirming that proliferation is a common event and does continue over many months. The low number of dividing cells at the time of removal in comparison to the total number of cells present, however, is an indicator that proliferation alone may not be responsible for the problems observed with the ERMs. Treatment strategies may need to take into consideration the timing of drug administration, as well as the contractile and possibly the inflammatory characteristics of the membranes to prevent the ensuing effects on the retina.

## Introduction

Epiretinal membranes (ERMs) are sheets of cells and extracellular matrix that occur on the vitreal surface of the retina. The most common are idiopathic ERMs (iERMs) [1], where there is no known underlying pathology; however, these can become clinically significant when they cause a decrease in visual acuity or metamorphopsia. ERMs also occur as a result of disease or trauma of the eye, such as in proliferative vitreoretinopathy (PVR), proliferative diabetic retinopathy (PDR), and after successful retinal detachment repair (ERMpRD). The latter occur in up to 6% of patients and behave differently from PVR membranes [2]. The different membrane types cause variable clinical symptoms, such as metamorphopsia, blurred vision, micropsia, or macropsia, and problems such as retinal traction or even traction retinal detachment. In all cases, however, ERMs appear to form initially as a result of cells from within the retina, such as retinal pigment epithelium (RPE), Müller cells, and astrocytes, that begin proliferating and migrating onto the surface of the retina. The cells can either migrate through a hole or tear in the retina, as is generally assumed to be the case for RPE cells, or they can simply extend processes out of the retina, as in the case of glial cells. Once this scaffold has formed, other cell types present at the vitreoretinal interface, such as hyalocytes and macrophages, may contribute to the cells undergoing proliferation. ERMs become a serious problem when they are contractile, often resulting in a folding or detachment of the retina. It has been postulated by Machemer [3] and MacLeod and colleagues [4,5] that the formation of ERMs is an aberrant form of healing response, with an initial proliferation phase, after which the contraction phase begins.

Determining the exact cell types present in the membranes has been the focus of several previous studies [4,6-12]. Using light or electron microscopy, it has been shown that many cells seem to change their morphological characteristics as the ERM develops, making it difficult to identify their origin [6-9]. In earlier studies [6,13,14], cells were identified as myofibroblasts, hyalocytes, fibrous astrocytes, RPE, and macrophages using only morphology as the means of identification. With the addition of immunocytochemistry, however, it has become apparent that the most obvious cell types involved are glia, macrophages, RPE cells, and fibroblasts [8,11,15,16]. Using antibodies to the protein K_i_-67 to label frozen and wax-embedded sections, Heidenkummer et al. [11,15] quantified levels of cell proliferation in different types of ERMs. They suggested that such data could help predict membrane behavior and thus be of clinical significance when trying to predict the risk of recurrence [11,15]. They developed a “proliferation index,” which is the total number of cells divided by the number of dividing cells. This index could help to clinically classify membrane types and “quantitatively indicate the proliferation potential of the ERM” [11,15]. However, the researchers did not correlate the proliferation index with the underlying disease process in these studies, as they felt their numbers were too low to separate the interindividual differences from the disease process differences [11,15].

To understand the origins of dividing cell types in four different ERM types we labeled whole membranes with antibodies to the K_i_-67 protein to detect proliferating cells, and antibodies specific to proteins for glial, immune, and RPE cells. We then correlated the number of dividing cells and the cell types to the type of ERM and the duration of the disease. We show that proliferating glial, RPE, and immune cells could be identified in all four types of ERMs, although the number of dividing cells varied both with disease type and the duration of the disease. Implications for treatment strategies are discussed.

## Methods

**Figure 1:**
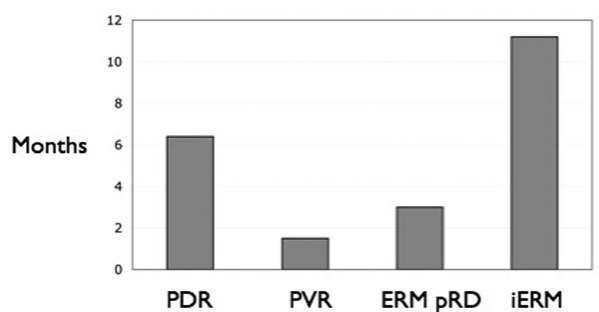
Graph illustrating the mean length of time, in months, between diagnosis and epiretinal membrane removal for each disease condition. The duration the membranes were present in the eye ranged from 2 to 14 months for proliferative diabetic retinopathy (PDR), 0.25- 6 for proliferative vitreoretinopathy (PVR), 1–5 for epiretinal membrane post–retinal detachment (ERMpRD), and 4–25 for idiopathic ERM (iERM).

Forty-six ERMs from male and female patients undergoing vitrectomy were obtained at the Academic Medical Center, Amsterdam, Netherlands. The ERMs were classified as follows: PDR (n=8), PVR (n=16), ERMpRD (n=5), and iERM (n=17). Upon removal from the eye, the membranes were immediately fixed in 4% paraformaldehyde in 0.1M sodium cacodylate buffer, pH 7.4 (Electron Microscopy Sciences, Fort Washington, PA) and stored at 4 °C until used. The period between the diagnosis of the condition and the actual surgery for the condition was between 1 and 25 months, i.e., some patients presented and had surgery within one month, but often ERMs were diagnosed clinically that did not require immediate surgery, as the patient was asymptomatic. The average duration (in months) that the membranes were present in the eye before removal was 6.5 (range 2–14) for PDR, 1 (range 0.25–6) for PVR, 3 (range 1–5) for ERMpRD, and 11 (range 4–25) for iERM (Figure 1). The iERM group presented the longest delay between diagnosis and removal of the membrane, since the membranes often showed no effect on vision at the time of diagnosis. It was more difficult to assess the duration of the PDR membranes. In this case, we used either a decrease in vision, indicating active neovascularization with traction or bleeding, or the first evidence of fibrovascular proliferation noted on clinical examination. All procedures had institutional research ethics committee approval and adhered to the tenets of the Declaration of Helsinki.

### Immunocytochemistry

ERMs were processed whole in 1.5 ml Eppendorf tubes (Fisher Scientific, Pittsburgh, PA) without embedding or sectioning. Following fixation, the tissue samples were rinsed three times for 5 min each, and then once for 1 h in phosphate buffer solution (PBS; 10× PBS stock: 250 ml distilled water, 21.92 g NaCl, 0.67 g NaH_2_PO_4_, 2.88 g Na_2_HPO_4_; stir well, dilute 1:10 in distilled water, adjust pH to 7.4. Yields 0.086 M PBS). Since the antibodies to K_i_-67 (e.g., anti-MIB-1 and anti-SP6) require antigen retrieval to expose the epitopes, the tissue was incubated in citrate buffer (Dako, Carpinteria, CA) for 40 min at 97 °C. Following cooling for 20 min at room temperature, the tissue was rinsed three times in PBS (for 250 ml, add 21.92 g NaCl, 0.67 g NaH_2_PO_4_, and 2.88 g Na_2_HPO_4_) for 5 min each time. The tissue was then incubated in 1:20 normal donkey serum in a mixture that contained PBS, 0.5% BSA (BSA), 0.1% Triton X-100 (Fisher Scientific, Pittsburgh, PA), and 0.1% azide (PBTA) overnight at 4 °C on a rotator. The following day, the primary antibodies were added to the PBTA and the tissues were again left overnight at 4 °C on a rotator. Next, either mouse monoclonal anti-MIB-1 (1:100; Immunotech/Beckman Coulter, Fullerton, CA) or rabbit polyclonal anti-SP6 (1:100; Abcam, Cambridge, MA) antibodies were used to label the K_i_-67 protein (referred to hereafter as K_i_-67 labeling for simplification). Two different markers were used to label K_i_-67 because of the limitations in triple label combinations. Anti-MIB-1 was used in combination with rabbit polyclonal antibodies to glial fibrillary acidic protein (GFAP; 1:400; Dako, Carpinteria, CA), which is an intermediate filament protein in glial cells, as well as biotinylated *Ricinus communis* (ricin; 1:1000; Vector Labs, Burlingame, CA), a lectin that labels immune cells such as microglia and macrophages [17]. Anti-SP6 was used in combination with antibodies to ezrin (mouse monoclonal; 1:10,000; Sigma, St. Louis, MO), a cytoskeletal protein in the microvilli of the RPE, and either vimentin (chicken monoclonal; 1:2,000; Chemicon, Temecula, CA), an intermediate filament protein present in glia, or biotinylated *Ricinus communis*. After rinsing of the primary antibodies in PBTA, the following secondary probes were added in PBTA, and the mixture rotated overnight at 4 °C: donkey anti-mouse conjugated to CY3 for MIB-1, donkey anti-rabbit conjugated to CY2 for GFAP, streptavidin CY5 for ricin, donkey anti-rabbit conjugated to CY3 for SP6, donkey anti-chicken conjugated to CY2 for vimentin, and donkey anti-mouse conjugated to CY2 for ezrin. All secondary antibodies were purchased from Jackson ImmunoResearch (West Grove, PA) and used at a concentration of 1:200. Streptavidin was purchased from Vector Labs (Burlingame, CA) and used at a concentration of 1:100. On the final day, the sections were rinsed in PBTA, after which 1:5,000 Hoechst stain (Invitrogen, Carlsbad, CA) was added to all samples for identification of cell nuclei. The membranes were mounted on glass slides in 5% n-propyl gallate in glycerol and viewed on an Olympus FluoView 500 laser scanning confocal microscope (New York, NY). Images were collected as a “z” series of 5 to 10 images taken at 0.5 µm intervals. For quantitation, a single plane image was selected from the “z” series that contained the greatest number of anti-K_i_-67 labeled nuclei.

Typically, 1 or 2 low magnification images (e.g., 200×) were taken of each membrane depending on its size. The number of anti-K_i_-67-labeled cells, as well as the total number of nuclei in the image plane were counted manually from the digital images and normalized to the area of tissue for each image, providing a population density (cells/mm^2^ hereafter simply referred to as “density”) for total nuclei and the anti-K_i_-67 labeled cells (Table 1). Labeling with the proliferating cell markers was correlated with the presence of glial, RPE, and immune cell markers, while images were viewed in Photoshop CS2 (Adobe, San Jose, CA), where magnification and channel (color) intensity could be adjusted. This ensured accuracy when determining which cell types were double labeled with the K_i_-67 markers.

**Table 1:** Table showing the quantification of all 46 ERMs from the 4 disease conditions.

				**Anti-K_i_-67 positive**				
**Membrane/type**	**Area (mm^2^)**	**Total nuclei**	**Nuclei/mm^2^**	**Glia/mm^2^**	**Immune/mm^2^**	**RPE/mm^2^**	**Unknown/mm^2^**	**Total K_i_-67/mm^2^**
**Figure 5**** PDR**								
1	0.5835	1970	3376	0	2		9	11
2	0.6436	1584	2461	2	0		12	14
3	0.1278	448	3505	0	23		0	23
4	0.4514	1657	3671	0	9		29	38
**Figure 6**** PDR**								
5	0.1557	66	424	0	0		19	19
6	0.3101	1365	4402	13	3		6	22
7	0.921	3467	3764	26		3	0	29
8	0.7658	1379	1801	1		3	7	11
**Figure 5**** PVR**								
9	0.5292	1762	3330	8	8		17	33
10	0.6947	2689	3871	6	3		35	44
11	0.3097	716	2312	0	3		29	32
12	0.398	2017	5068	10	85		405	500
13	0.6892	3091	4485	0	1		39	40
14	0.4834	2301	4760	17	6		27	50
15	0.4648	2414	5194	103	15		60	178
16	0.0804	536	6667	12	25		50	87
17	0.2633	888	3373	8	15		15	38
18	0.3426	920	2685	88	6		47	141
19	0.1111	469	4221	27	0		45	72
20	0.3293	1316	3996	9		12	0	21
21	0.3627	1596	4400	8		25	0	33
**Figure 6**** PVR**								
22	0.3256	1895	5820	0		3	0	3
23	0.7879	3231	4101	0		203	0	203
24	0.1112	1182	10629	162		558	18	738
**Figure 5**** ERMpRD**								
25	0.2172	478	2201	0	18		0	18
26	0.7095	3386	4772	0	0		6	6
27	0.3297	983	2981	0	0		3	3
**Figure 6**** ERMpRD**								
28	0.2892	1154	3990	0		9	0	14
29	0.2002	789	3941	0		10	15	20
**Figure 5**** iERM**								
30	0.9827	4500	4579	2	1		5	8
31	0.2196	264	1202	5	0		0	5
32	0.6483	791	1220	6	0		3	9
33	1.0166	1734	1706	1	0		11	12
34	0.9437	2619	2775	7	5		5	17
35	0.1648	481	2919	6	0		55	61
36	0.1481	384	2593	7	0		7	14
37	0.1615	471	2916	31	6		19	56
38	0.1615	753	4663	19	6		6	31
39	0.2046	1120	5474	10	0		0	10
40	0.6502	1028	1581	12		26	0	38
41	0.7759	1317	1697	3		0	3	6
42	0.7448	397	533	8		0	8	16
**Figure 6**** iERM**								
43	0.6299	1159	1840	6		0	6	12
44	0.448	631	1408	9		0	4	13
45	0.5551	780	1405	11		0	4	15
46	0.7505	1067	1422	1		0	4	5

The raw data are shown to illustrate the similarities and differences between each membrane. The last column for Total K_i_-67+ cells/mm^2^ is plotted in Figure 4 as an average of these numbers which also shows the standard deviation for each disease group. The figure numbers at the left of the table indicate which group of membranes were used to generate Figure 5 and Figure 6.

Using antibodies to K_i_-67 protein to identify proliferating cells in histopathology specimens has several strengths: 1) the antibodies will label dividing cells in all phases of the cell cycle except G0 (resting) [18-20]; 2) they do not require living tissue, as does commonly used ^3^H-thymidine labeling; and 3) the antibodies allow relatively straightforward labeling with multiple probes for the identification of different cell types.

## Results

### Immunocytochemistry

**Figure 2:**
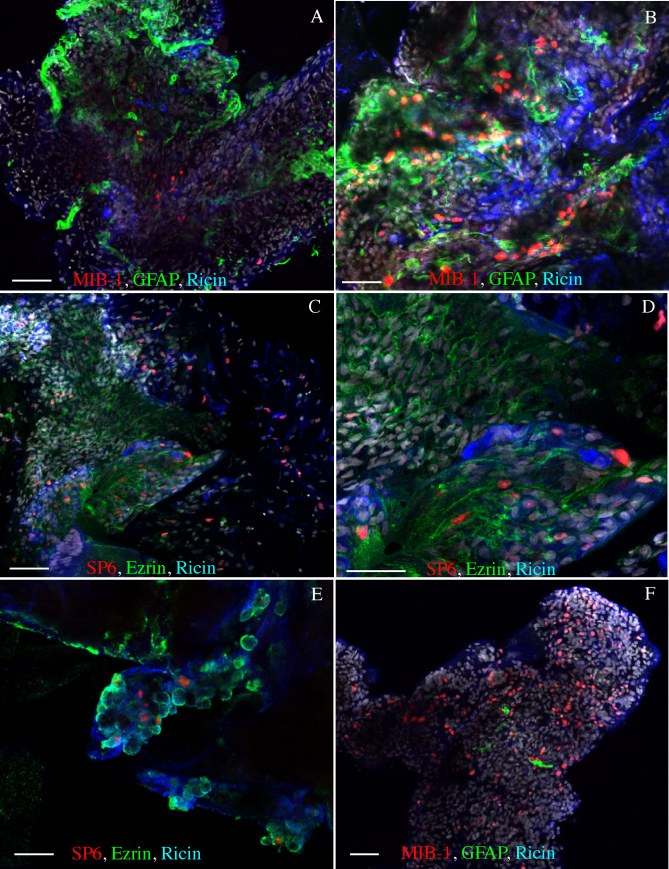
Images of representative staining patterns on epiretinal membranes from proliferative vitreoretinopathy. Anti-MIB-1 (**A**, **B**, **F**; red) or anti-SP6 (**C**, **D**, **E**; red) labeling was observed among all cell types: glia (**A**, **B**, **F**; green), immune cells (**A**-**F**; blue) retinal pigment epithelial (RPE) cells (**C**, **D**, **E**; green). Note that the amount of anti-glial fibrillary acidic protein (GFAP) labeled glia varied between membranes (**A**, **B**, **F**). The anti-ezrin labeling appeared to encircle the cells, and the ricin labeling was prevalent in all samples. Scale bars equal 50 µm.

**Figure 3:**
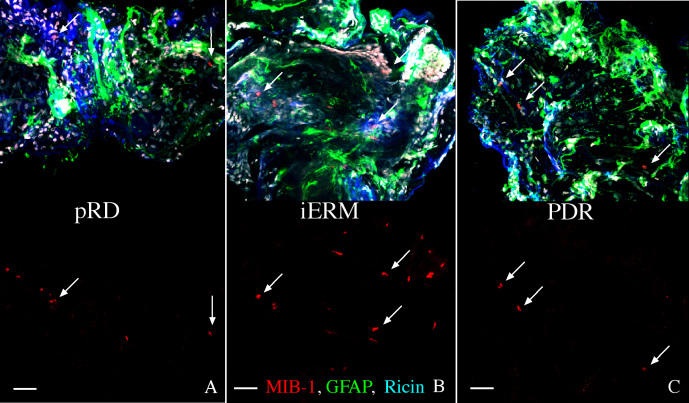
Images of representative staining patterns on three different types of epiretinal membranes. The membranes were from post-retinal detachment (pRD; **A**), of idiopathic origin (iERM; **B**), and proliferative diabetic retinopathy (PDR; **C**). Each membrane is labeled with anti-MIB-1 (red), anti-glial fibrillary acidic protein (GFAP; green) and ricin (blue). In the lower half of each image the green and blue channels are turned off to more easily see the anti-MIB-1 staining (arrows are for reference points). Scale bars equal 50 µm.

Anti-K_i_-67 (MIB-1, SP6) was used to identify proliferating cells, anti-ezrin to identify RPE cells, anti-GFAP or anti-vimentin to identify glial cells, ricin to identify immune cells, and Hoechst staining used to identify all nuclei (Figure 2 and Figure 3). However, since only four fluorochromes could be used at one time, and not all antibodies could be used in combination because they were made in the same species, the membranes were divided into labeling groups: 1) anti-K_i_-67+anti-GFAP+ricin+Hoechst, 2) anti-K_i_-67+anti-vimentin+anti-ezrin+ Hoechst, or 3) anti-K_i_-67+anti-ezrin+ricin+Hoechst. Each group contained some anti-K_i_-67-labeled cells not labeled with any cell-specific marker. These are listed as “unidentified” in the tabulated data (Table 1), and cells could fall into this category because they were truly not recognized by any of the markers we used or because their specific marker was not included in the marker combination used to stain that specific membrane, i.e., RPE cells in the first group, immune cells in the second group, or glial cells in the third group.

All membranes were found to be multilayered and highly cellular, as illustrated by the Hoechst nuclear stain (Figure 2, Figure 3; white nuclei). While anti-K_i_-67 labeled nuclei formed only a small part of this total population, they occurred in all ERMs examined. In addition, all ERMs had cells labeled with the markers for glial, immune (microglia and macrophages), and RPE cells; and examples from all of these cell types were anti-K_i_-67 labeled across the different membranes. Indeed, most of the anti-K_i_-67-labeled cells were also labeled with one of the cell-specific antibodies used here (Table 1). Examples of typical staining patterns observed in six different membranes from the PVR group are illustrated in Figure 2. In general, most PVR membranes were richly populated with glial and RPE cells, which usually appeared clustered in separate domains, whereas the immune cells usually appeared dispersed throughout. In rare instances, the PVR membranes had only a few widely dispersed cells stained with a glial cell marker (Figure 2F) In addition, while anti-GFAP staining appeared to fill the cytoplasm of the glial cells (Figure 2A,B and Figure 3A-C), anti-ezrin staining appeared to encircle the RPE cells, (Figure 2C-E). In situ, anti-ezrin labels the apical microvilli of RPE cells; however, within the ERMs, the RPE cells did not exhibit this type of polarity.

Examples of common staining patterns from pRD, iERM, and PDR membranes labeled with anti-K_i_-67 (MIB-1), anti-GFAP, and ricin are shown in Figure 3A-C. The MIB-1 labeling alone is shown in the bottom half of each image to allow better visualization of the dividing cells (arrows are placed for reference points). All three samples contained dividing cells as well as glia and immune cells. Albeit at low levels by comparison to PVR membranes, anti-ezrin labeling of RPE cells was in fact also observed in pRD (in two out of two membranes), PDR (in two out of two membranes), and iERM membranes (in one out of seven membranes; Table 1).

### Quantitation

All 46 membranes were highly cellular, as determined by Hoechst staining (Table 1; “total nuclei/mm^2^”), far more than were identified by the anti-K_i_-67 antibodies. All membranes from the four disease conditions did, however, contain some dividing cells (Table 1).

**Figure 4:**
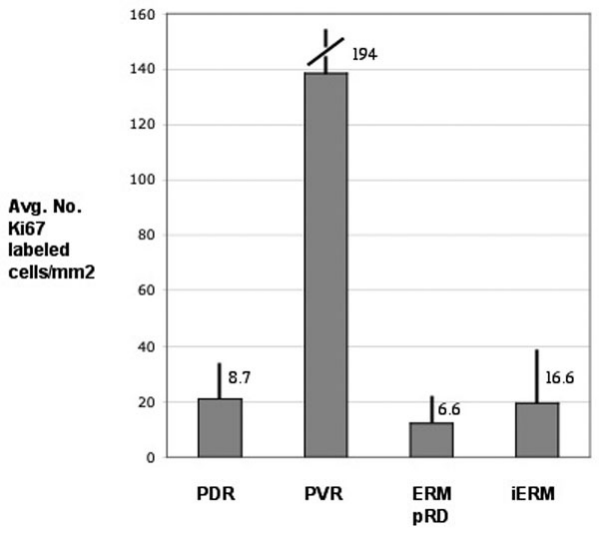
The average number of K_i_-67 labeled cells/mm^2^ of membrane for the four disease conditions plotted from the total K_i_-67/mm^2^ data in Table 1. PVR membranes had the highest number of dividing cells at 138.3/mm^2^, with proliferative diabetic retinopathy (PDR) at 20.9, epiretinal membranes post–retinal detachment (ERMpRD) at 12.2, and idiopathic ERM (iERM) at 19.3. Error bars represent the standard deviation.

The last column in Table 1 for total K_i_-67+ cells/mm^2^ is presented graphically as the average number of dividing cells in Figure 4, which also shows the standard deviation for each disease group. This illustrates that PVR membranes were clearly more active in terms of cell division than any of the other three disease conditions. The variability (standard deviation, Figure 4) is very large for each group but this is an accurate representation of the great variability observed between the different membranes.

**Figure 5:**
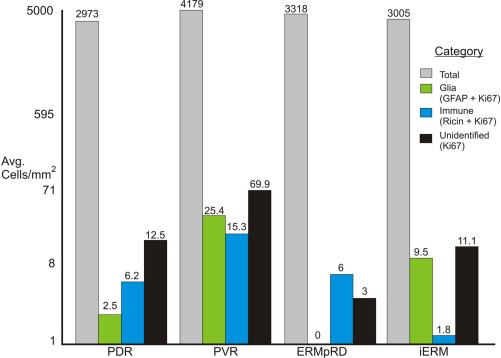
Illustration showing the average number of cells/mm^2^ for all four types of epiretinal membranes. The brackets at the left of Table 1 show which membranes were used to generate this figure. For each membrane type (i.e., disease condition) the average number of cells/mm^2^ equals the total number of nuclei/mm (gray bars) divided by the number of epiretinal membranes (ERMs) in the group. The average number of glial cells (green bars) equals the number of glial fibrillary acidic protein (GFAP) positive cells that were also labeled with K_i_-67/mm^2^ in each ERM divided by the number of ERMs in the group. The value for immune cells (ricin labeled cells, blue bars) was calculated the same way while “unidentified” equals the value for cells that were K_i_-67 positive but not labeled with any other markers (black bars). Proliferative vitreoretinopathy (PVR) membranes had the highest number of dividing cells. Abbreviations: proliferative diabetic retinopathy (PDR); proliferative vitreoretinopathy (PVR); post–retinal detachment (ERMpRD); idiopathic ERM (iERM).

**Figure 6:**
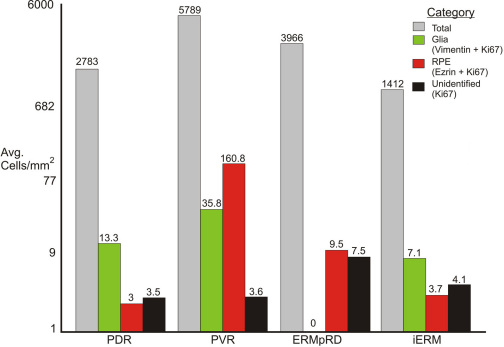
Illustration showing the average number of cells/mm^2^ for all four types of epiretinal membranes. The brackets at the left of Table 1 show which membranes were used to generate this figure. For each membrane type (i.e., disease condition), the average number of cells/mm^2^ equals the total number of nuclei /mm (gray bars) divided by the number of epiretinal membranes (ERMs) in the group. The average number of glial cells (green bars) equals the number of vimentin-positive cells that were also labeled with K_i_-67/mm^2^ in each ERM divided by the number of ERMs in the group. The value for retinal pigment epithelium (RPE) cells (ezrin labeled cells, red bars) was calculated the same way, while “unidentified” equals the value for cells that were K_i_-67 positive but not labeled with any other markers (black bars). Abbreviations: proliferative diabetic retinopathy (PDR); proliferative vitreoretinopathy (PVR); post–retinal detachment (ERMpRD); idiopathic ERM (iERM).

The relative proportion of the different K_i_-67 positive cell types (i.e., glia, RPE, immune, and unidentified) in the different classes of ERMs is shown in Figure 5 and Figure 6. (The brackets at the left of Table 1 show which membranes were used to generate Figure 5 and Figure 6.) While useful for illustrative purposes, the small number of samples in some categories (Table 1) precluded the statistical analysis of this data. Nevertheless, the graphs illustrate several interesting trends in the data. First, while all membranes contained many nuclei (gray bars, Figure 5 and Figure 6), only a small number of these cells were K_i_-67 labeled. Second, active glial cell proliferation was observed in all groups except for the ERMpRD group, although antivimentin or anti-GFAP labeling demonstrated the presence of glial cells suggesting that much of glial proliferation occurred earlier in the course of membrane formation. Third, K_i_-67 labeled cells of presumed immune origin (ricin-positive) were found in all four disease groups, indicating an inflammatory component to ERM formation regardless of the disease condition. Fourth, K_i_-67 labeled RPE cells were detected in the PDR and iERM groups suggesting that even without a tear or break in the retina, RPE cells can contribute to ERM formation.

An overall summary of the data are shown in Table 2 where the four disease conditions are compared in terms of 1) disease duration, 2) average size of the ERM, 3) average number of nuclei, and 4) average number of K_i_-67 positive cells. From this table, it can be seen that while PVR membranes were in the eye for the shortest duration (1.5 months), they had the highest proliferation rates and greatest density of nuclei. The iERM group had the longest duration, with an average of 11.2 months from the start of clinical symptoms, and had the largest average area, but were the most sparsely populated with nuclei. Finally, although the duration for ERMpRD, PDR and iERM ranged from 3 to 11 months, the average number of K_i_-67 cells was similar among them, indicating perhaps that a relatively constant low level of proliferation occurs throughout the time these membranes are present in the eye.

**Table 2:** Table showing a summary of the data collected from the 4 disease conditions.

**Parameters**	**PDR**	**PVR**	**ERM pRD**	**iERM**
Avg disease duration, months	6.4	1.5	3	11.2
Avg area of ERM (mm^2^)	0.49	0.39	0.35	0.54
Avg No. nuclei / mm^2^	2925	4682	3577	2349
Avg No. K_i_-67 cells / mm^2^	20.9	138.3	12.2	19.3

The average duration the membranes were in the eye before removal is related to the average area of the membrane, number of total nuclei and number of total K_i_-67 positive cells. Note that proliferative vitreoretinopathy (PVR) membranes were present in the eye for the shortest duration but had the most nuclei and divding cells whereas idiopathic epiretinal membrane (iERMs), which were present in the eye for the longest duration had fewer nuclei and divding cells compared to PVR.

## Discussion

Antibodies to the K_i_-67 protein are routinely used to show DNA synthesis and presumed cell division [18-20]. In previous studies of proliferation in ERMs removed from human patients, these antibodies have only been used on histological sections [11,15]. Our study, however, presents unique data from studying proliferation in whole mounts of ERMs by confocal imaging, thus providing a more complete picture of the total number of nuclei and the number of cell types undergoing proliferation at the time of removal from the eye. Using anti-K_i_-67 labeling in combination with cell-type specific markers, we showed the presence of proliferating glial, RPE, and immune cells in all four types of ERM studied here, regardless of their duration in the eye. Although we could not use all of the markers on each ERM, our general impression is that most cells in the ERMs fell into one of these three classes. Based on previous attempts to identify cells within human ERMs, it is likely that fibroblasts, and immune cells not labeled by the lectin ricin make up a minor component of the membranes and are included in our “unidentified” category, although further study with specific probes is required to confirm this conclusion.

While all membranes contained evidence for active cell proliferation, the amount varied depending upon disease condition. We observed that ERMs clinically assumed to be rapidly growing (PVR), had both the greatest density of cells and the greatest number of anti-K_i_-67 labeled cells. These results are in agreement with previous data reported from histologically sectioned membranes [11,12,15,21,22]. Thus proliferation is most likely a major contributor to the rapid expansion of PVR membranes. In this study, the membranes assumed to be “older” (e.g., iERMs) had a much lower level of K_i_-67 labeling, indicating that they may be less “reactive” in terms of cellular proliferation. This is in contrast to results reported by Zhang et al. [12], in which they noted no difference in proliferation rates between membrane types. There are two possible reasons for this difference: The previous study included only two membranes classified as “idiopathic,” and they used histological sections instead of whole mounts. The low numbers of proliferating cells that may be encountered in these membranes, along with the large variability we observed, clearly demonstrates the need for large sampling procedures in a study such as this.

The combined use of markers for all nuclei and those specific to different cell types revealed several new findings. We showed that these membranes are densely populated with nuclei (ranging at the high end between more than 3,000 to 10,000 nuclei/mm^2^), indicating that they are much more “cellular” than previously thought [8,11,15]. Indeed, this is the first study to attempt to describe the overall cellular composition in individual ERMs using whole mounts instead of histological sections, and the first to use Hoechst staining as a means for estimating the number of nuclei present. Our immunostaining data indicated that the greatest accumulation of proliferating RPE cells occurred in PVR and ERMpRD, with many fewer RPE cells in the PDR and iERM membranes. We expected to find RPE cells in the PVR and ERMpRD group, since the presence of retinal tears provides a passage for these cells across the retina [6,14]. Interestingly, however, the eyes with PDR and iERM membranes had no history of retinal tears either before or during surgery, but did show evidence of the presence of dividing RPE cells. These data strongly support the possibility that RPE cells can migrate through the intact retina [23,24]. We also found that while glial cells were present in all membrane types, there was no evidence for dividing glia in the ERMpRD group. This suggests that a rapid phase of proliferation may have ended early after detachment and what we observed in the membranes preserved for this study are results of sampling after this early proliferative phase, the expansion of cells residing in the membrane, and the growth of processes from glial cells in which the cell body remains within the retina. Indeed, following experimental retinal detachment in animal models, most of the observed proliferation occurs in Müller cells within the inner nuclear layer during the first three to four days after detachment [25,26]. As detachment (or reattachment) time increases, these cells grow and expand along the retinal surface [27]. In later stages of ERM formation, it may be contraction rather than proliferation that plays the key role in causing wrinkling and subsequent redetachment of the retina. There also appears to be a significant cellular inflammatory component in all ERMs, as shown by the ricin labeling. The presence of macrophages, lymphocytes, and monocytes in ERMs has been noted previously; however, there has been no quantification of cells involved in this response [28-32]. It is not clear whether immune activation is in response to the initial (micro) trauma of the disease or posterior vitreous detachment [33] or if it is a self-perpetuating reaction, possibly caused by a breakdown of the blood-retina barrier. However, the immune cells we observed were not only found in all membranes, but were also actively proliferating in all membrane types.

A parallel between wound healing and ERM formation has been postulated previously. Our data are generally consistent with this hypothesis. ERM formation probably involves an early proliferative phase with a higher cell division rate and a slower cell division rate in the older, less reactive membranes. After the proliferative phase, there is a contractile phase with the deposition and contraction of extracellular matrix [2,3]. However, it was somewhat surprising that ERMs during what would be clinically described as the active phase of PVR showed less cell proliferation than might be expected. This suggests that perhaps much of the cellular proliferation is a very early event in membrane formation and indeed may even occur within the retina before the actual formation of clinically observable membranes. By the time these membranes are surgically removed, they have entered the secondary phase of extracellular matrix deposition and contraction. This would imply that clinical treatments aimed against membrane formation would need to be given as early as possible. Once the membrane has formed, anticontractile agents may be more effective than antiproliferative drugs.

In conclusion, by using whole mounts of ERMs from different disease conditions combined with nuclear staining we have shown that the cellularity of ERMs is higher than previously shown in studies that relied on histological sections. The whole mounts clearly show that ERMs are composed of areas rich in cells alongside areas that are mostly composed of extracellular matrix. By combining anti-K_i_-67 antibodies with cell-specific markers we have identified various cell types proliferating in the different types of ERM, and find that the proportions differ among diseases. We also showed that the proliferation rate is higher in membranes of shorter duration, that is, those removed from the eye early because it is assumed that the ERM is highly reactive. While this may not be surprising, we have added new information by providing estimates of the number of dividing cells for each disease condition. Finally we show that in conditions where there is no tear or hole in the retina, RPE cells can be observed in the ERMs. The fact that the overall numbers of dividing cells were relatively low compared to the total number of nuclei in the membranes suggests that much of the proliferation may occur early, before the actual clinical manifestations, which ultimately may have significant implications for timing of therapeutic and clinical interventions.

## References

[1] McCarty DJ, Mukesh BN, Chikani V, Wang JJ, Mitchell P, Taylor HR, McCarty CA (2005). Prevalence and associations of epiretinal membranes in the visual impairment project.. Am J Ophthalmol.

[2] Uemura A, Ideta H, Nagasaki H, Morita H (1992). Ito k. Macular pucker after retinal detachment surgery.. Ophthalmic Surg.

[3] Machemer R (1977). Massive periretinal proliferation: a logical approach to therapy.. Trans Am Ophthalmol Soc.

[4] Hiscott PS, Grierson I, McLeod D (1985). Natural history of fibrocellular epiretinal membranes: a quantitative, autoradiographic, and immunohistochemical study.. Br J Ophthalmol.

[5] Gilbert C, Hiscott P, Unger W, Grierson I, McLeod D (1988). Inflammation and the formation of epiretinal membranes.. Eye (Lond).

[6] Kampik A, Kenyon KR, Michels RG, Green WR, de la Cruz ZC (1981). Epiretinal and vitreous membranes, a comparative study of 56 cases.. Arch Ophthalmol.

[7] Morino I, Hiscott P, McKechnie N, Grierson I (1990). Variation in epiretinal membrane components with clinical duration of proliferative tissue.. Br J Ophthalmol.

[8] Vinores SA, Campochiaro PA, Conway BP (1990). Ultrastructural and electron-immunocytochemical characterization of cells in epiretinal membranes.. Invest Ophthalmol Vis Sci.

[9] Baudouin C, Brignole F, Bayle J, Fredj-Reygrobellet D, Lapalus P, Gastaud P (1991). Class II histocompatibility antigen expression by cellular components of vitreous and subretinal fluid in proliferative vitreoretinopathy.. Invest Ophthalmol Vis Sci.

[10] Heidenkummer HP, Kampik A (1991). Immunohistochemical localisation of epidermal growth factor receptor in human epiretinal membrane.. Graefes Arch Clin Exp Ophthalmol.

[11] Heidenkummer HP, Kampik A (1992). Proliferative activity and immunohistochemical cell differentiation in human epiretinal membranes.. Ger J Ophthalmol.

[12] Zhang X, Barile G, Chang S, Hays A, Pachydaki S, Schiff W, Sparrow J (2005). Apoptosis and cell proliferation in proliferative retinal disorders: PCNA, Ki-67, caspase-3, and PARP expression.. Curr Eye Res.

[13] Van Horn DL, Aaberg TM (1977). 344 Machemer R, Fenzl R. Glial cell proliferation in human retinal detachment with massive periretinal proliferation.. Am J Ophthalmol.

[14] Machemer R, van Horn D, Aaberg TM (1978). Pigment epithelial proliferation in human retinal detachment with massive periretinal proliferation.. Am J Ophthalmol.

[15] Heidenkummer HP, Kampik A, Petrovski B (1992). Proliferative activity in epiretinal membranes. The use of the monoclonal antibody Ki-67 in proliferative vitreoretinal diseases.. Retina.

[16] Jerdan JA, Pepose JS, Michels RG, Hayashi H, de Bustros S, Sebag M, Glaser BM (1989). Proliferative vitreoretinopathy membranes. An immunohistochemical study.. Ophthalmology.

[17] Lewis GP, Sethi CS, Carter KM, Charteris DG, Fisher SK (2005). Microglial cell activation following retinal detachment: a comparison between species.. Mol Vis.

[18] Gerdes J, Schwab U, Lemke H, Stein H (1983). Production of a mouse monoclonal antibody reactive with a human nuclear antigen associated with cell proliferation.. Int J Cancer.

[19] Gerdes J, Lemke H, Baisch H, Wacker HH, Schwab U, Stein H (1984). Cell cycle analysis of a cell proliferation-associated human nuclear antigen defined by the monoclonal antibody Ki-67.. J Immunol.

[20] Schlüter C, Duchrow M, Wohlenberg C, Becker MH, Key G, Flad HD, Gerdes J (1993). The cell proliferation associated antigen of antibody Ki-67: A very large, ubiquitous nuclear protein with numerous repeated elements, representing a new kind of cell cycle maintaining proteins.. J Cell Biol.

[21] Tsanou E, Ioachim E, Stefaniotou M, Gorezis S, Charalabopoulos K, Bagli H, Peschos D, Psilas K, Agnantis NJ (2005). Immunohistochemical study of angiogenesis and proliferative activity in epiretinal membranes.. Int J Clin Pract.

[22] Ioachim E, Stefaniotou M, Gorezis S, Tsanou E, Psilas K, Agnantis NJ (2005). Immunohistochemical study of extracellular matrix components in epiretinal membranes of vitreoproliferative retinopathy and proliferative diabetic retinopathy.. Eur J Ophthalmol.

[23] Hamilton CW, Chandler D, Klintworth GK, Machemer R (1982). A transmission and scanning electron microscopic study of surgically excised preretinal membrane proliferations in diabetes mellitus.. Am J Ophthalmol.

[24] Hiscott P, Gray R, Grierson I, Gregor Z (1994). Cytokeratin-containing cells in proliferative diabetic retinopathy membranes.. Br J Ophthalmol.

[25] Fisher SK, Erickson PA, Lewis GP, Anderson DH (1991). Intraretinal proliferation induced by retinal detachment.. Invest Ophthalmol Vis Sci.

[26] Geller SF, Lewis GP, Anderson DH, Fisher SK (1995). Use of the MIB-1 antibody for detecting proliferating cells in the retina.. Invest Ophthalmol Vis Sci.

[27] Fisher SK, Lewis GP, Lindberg KA, Verardo MR (2005). Cellular remodeling in mammalian retina: results from studies of experimental retinal detachment.. Prog Retin Eye Res.

[28] Baudouin C, Gordon WC, Fredj-Reygrobellet D, Baudouin F, Peyman G, Gastaud P, Bazan NG (1990). Class II antigen expression in diabetic preretinal membranes.. Am J Ophthalmol.

[29] Baudouin C, Fredj-Reygrobellet D, Gordon WC, Baudouin F, Peyman G, Lapalus P, Gastaud P, Bazan NG (1990). Immunohistologic study of epiretinal membranes in proliferative vitreoretinopathy.. Am J Ophthalmol.

[30] Charteris DG, Hiscott P, Grierson I, Lightman SL (1992). Proliferative vitreoretinopathy. Lymphocytes in epiretinal membranes.. Ophthalmology.

[31] Charteris DG, Hiscott P, Robey HL, Gregor ZJ, Lightman SL, Grierson I (1993). Inflammatory cells in proliferative vitreoretinopathy subretinal membranes.. Ophthalmology.

[32] Tang S, Scheiffarth O, Thurau SR, Wildner G (1993). Cells of the immune system and their cytokines in epiretinal membranes and in the vitreous of patients with proliferative diabetic retinopathy.. Ophthalmic Res.

[33] Foos RY (1977). Vitreoretinal juncture; epiretinal membranes and vitreous.. Invest Ophthalmol Vis Sci.

